# Supplementary material for: Ultrasonic barrier-through imaging by Fabry-Perot resonance-tailoring panel
Source: Nat Commun. 2023 Nov 28;14:7818. doi: 10.1038/s41467-023-43675-4 (PMC10684589; doi:10.1038/s41467-023-43675-4)
Supplement: Supplementary file 1 — Supplmentary Information file [file 41467_2023_43675_MOESM1_ESM.pdf]

<u>**Supplementary Information**</u>

Ultrasonic barrier-through imaging by Fabry-Perot resonance-tailoring panel

Chung Il Park[1,2], Seungah Choe[1,2], Woorim Lee[1,2], Wonjae Choi[3,4], Miso Kim[5,6], Hong Min Seung[3,4,*], and Yoon Young Kim[1,2,*]

[1]*Department of Mechanical Engineering, Seoul National University, 1 Gwanak-ro, Gwanak-gu, Seoul, 08826, Republic of Korea*

[2]*Institute of Advanced Machines and Design, Seoul National University, 1 Gwanak-ro, Gwanak-gu, Seoul, 08826, Republic of Korea*

[3]*Intelligent Wave Engineering Team, Korea Research Institute of Standards and Science (KRISS), 267 Gajeong-ro, Yuseong-gu, Daejeon, 34113, Republic of Korea*

[4]*Department of Precision Measurement, University of Science and Technology (UST), 217 Gajeong-ro, Yuseong-gu, Daejeon, 34113, Republic of Korea*

[5]*School of Advanced Materials Science and Engineering, Sungkyunkwan University (SKKU), 2066 Seobu-ro, Jangan-gu, Suwon, 16419, Republic of Korea*

[6] *SKKU Institute of Energy Science and Technology (SIEST), Sungkyunkwan University (SKKU), 2066 Seobu-ro, Jangan-gu, Suwon 16419, Republic of Korea*

This Supplementary Information file includes:
Supplementary Figs. 1-18, Supplementary Tables 1-6, and Supplementary Notes 1-5

Supplementary Figures

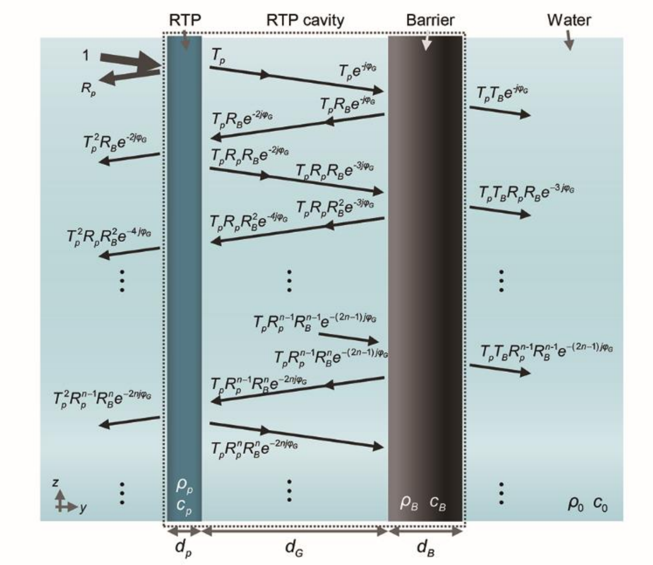

Supplementary Figure 1 | Multiple scattering analysis of RTP cavity. A train of wave reflections and transmissions in the case when both the RTP and the barrier are in the background medium (water). The transmission and reflection coefficients associated with the RTP and barrier are denoted by T_i and R_i, respectively (i=p, B). d_m (m= p, G, B) represents the barrier thickness. Subscript p, G, and B respectively denote the panel, gap, and barrier. j is the imaginary unit. ρ_0 and c_0 respectively denote the density and phase velocity of the water.

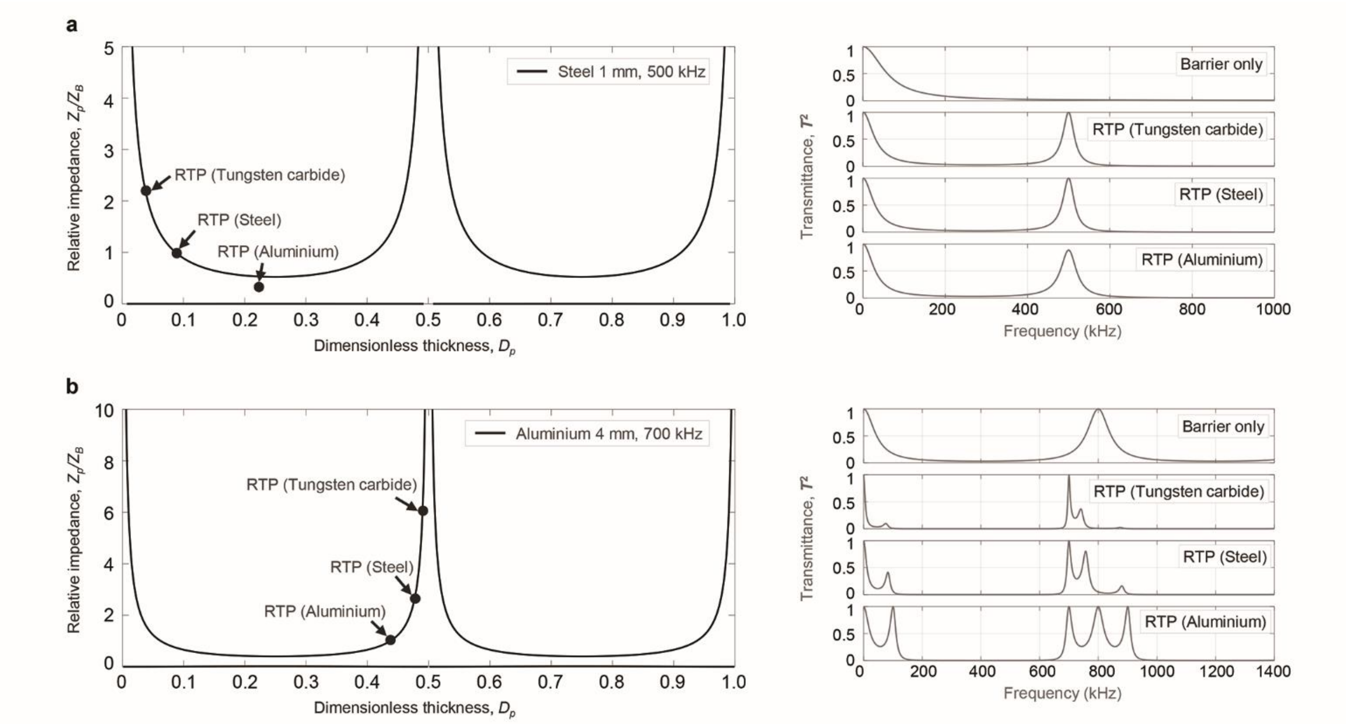

Supplementary Figure 2 | RTP design examples and their transmittance. A curve (right) used to determine D_p and Z_r of the RTPs considering three possible material candidates (tungsten carbide, steel, and aluminium). The corresponding transmittance curves (left) with and without the RTP as a function of frequency for **a**, a 1-mm-thick steel at 500 kHz and **b**, a 4-mm-thick aluminium at 700 kHz. The determined parameters of the RTPs are given in Supplementary Table 1.

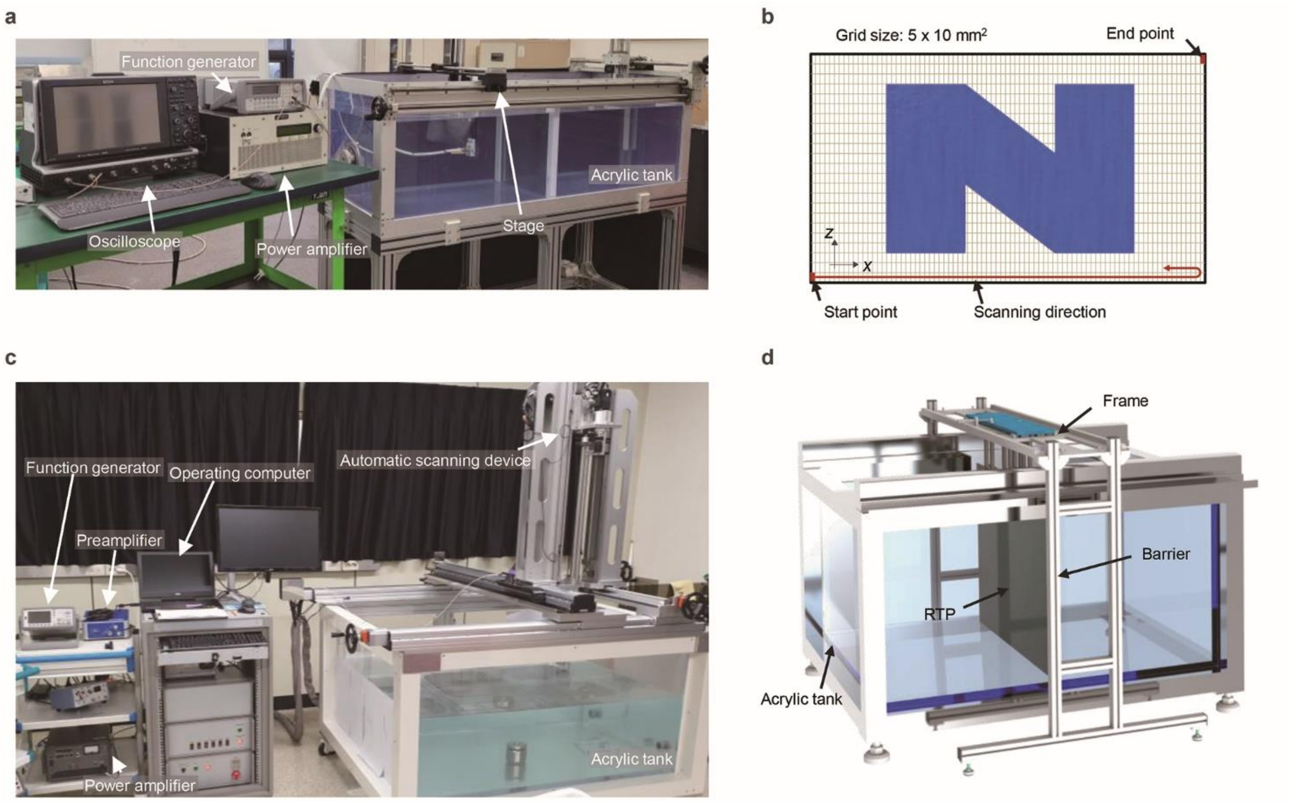

Supplementary Figure 3 | Experimental setup. a, A photograph of the water tank and devices used in the experiments using a 1-mm-thick steel plate barrier. **b**, A mesh used to scan the object. **c**, A photograph of the water tank and the automatic scanning device used in the experiments using a 4-mm-thick aluminium plate barrier. **d**, A schematic drawing showing the locations of the RTP and barrier. A frame was used to hold the RTP and barrier in place. This frame configuration was drawn by SOLIDWORKS and rendered by KEYSHOT 10.

,

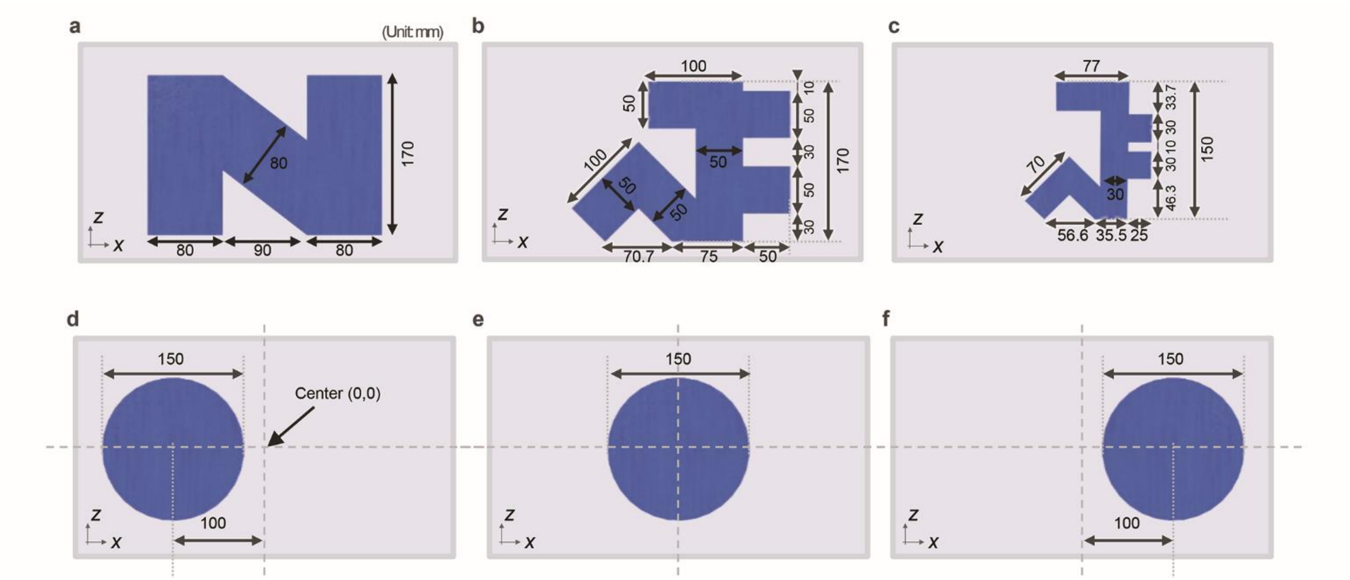

Supplementary Figure 4 | Object information. a-f, Geometric specifications of the objects. **a-c**, The objects for shape recognition tests. **d-f**, The objects for location estimation tests. The detailed specifications of the barrier and RTP including the material properties and thicknesses are listed in Supplementary Table 2.

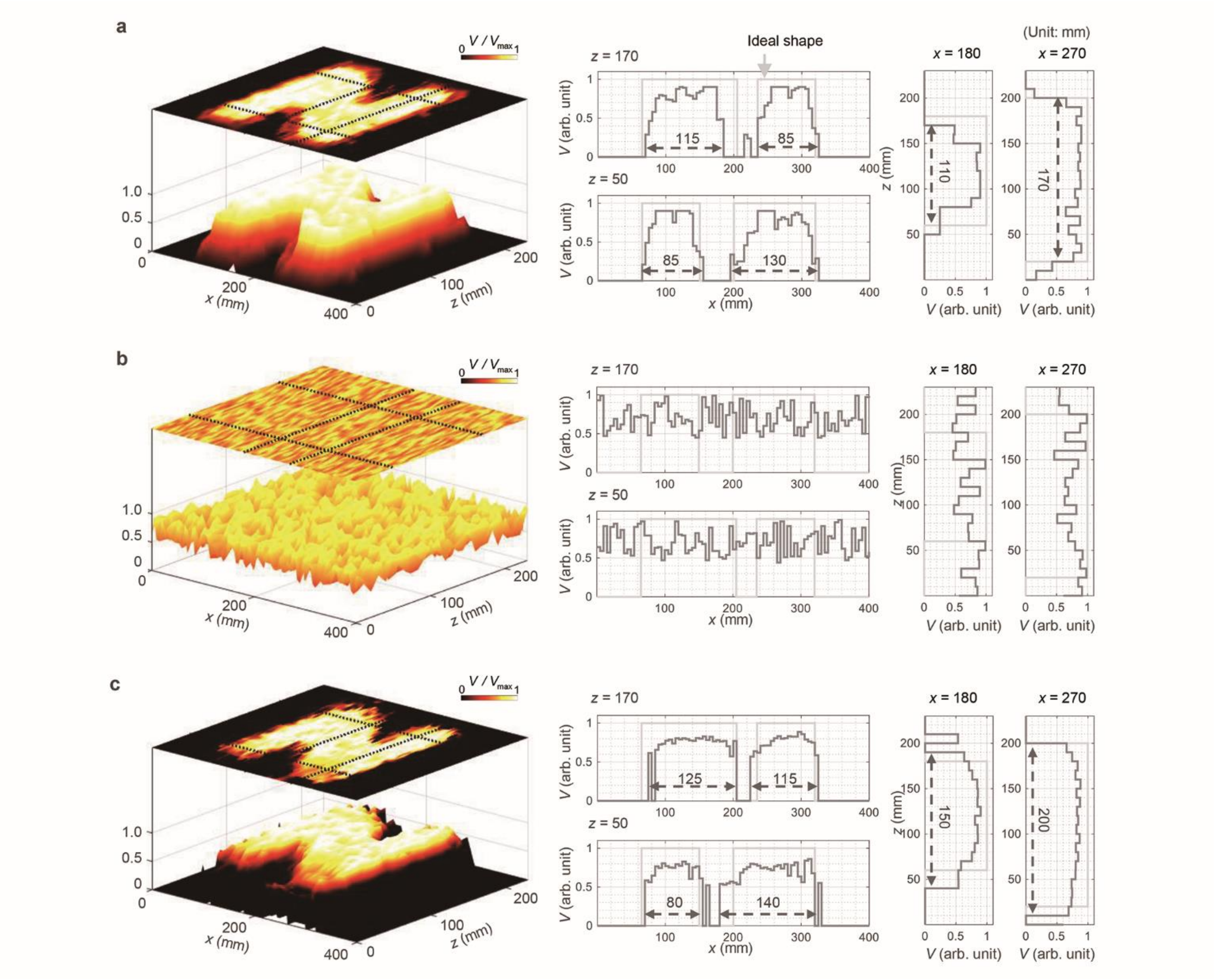

Supplementary Figure 5 | Processed images for the *N*-shaped object. The C-scanned image and the B-scan contours at z=50,170 mm and x=180, 270 mm for **a**, a case with no barriers, **b**, a case with the barrier only, and **c**, a case with the barrier and RTP.

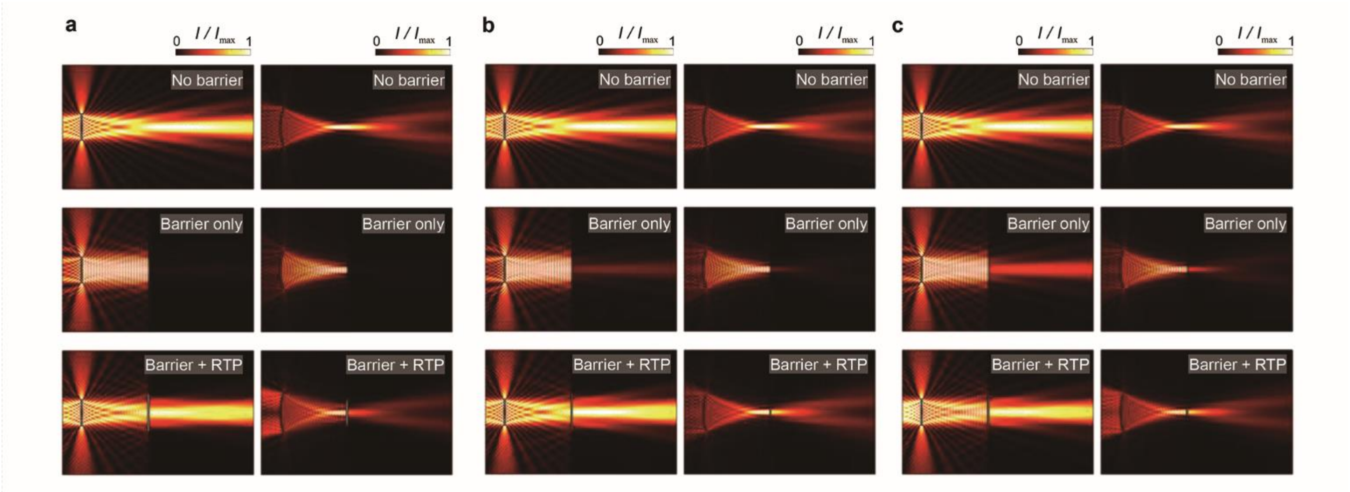

Supplementary Figure 6 | Beam transmission simulation with and without the RTP for different barrier types. Intensity fields of the ultrasound beam simulations regarding **a**, a 1-mm-thick steel barrier, **b**, a 1-mm-thick aluminium barrier, and **c**, a 1-mm-thick skull barrier. In **a-c**, (up): without barrier, (middle): with barrier only, (down): with the barrier and RTP. The barrier and RTP specifications and quantitative values of simulation results are listed in Supplementary Table 4.

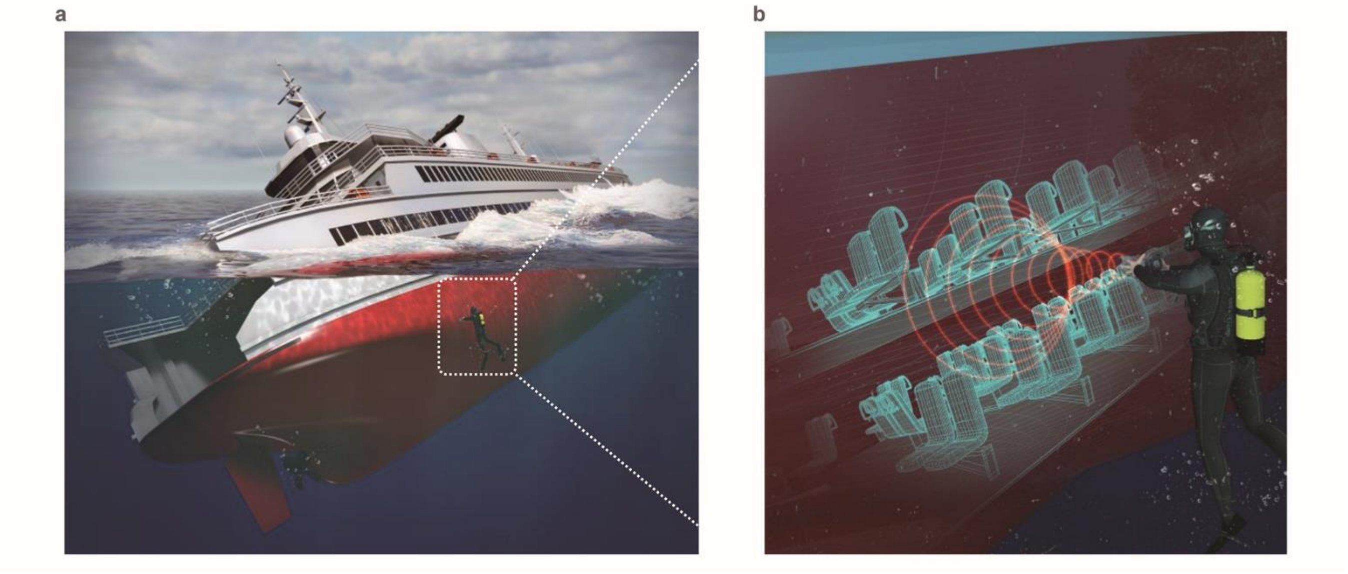

Supplementary Figure 7 | Research objectives and the current development. a, A schematic drawing of a vessel accident and **b**, RTPs-based barrier-through ultrasonic imaging for the urgent rescue. This figure was drawn by Adobe Illustrator.

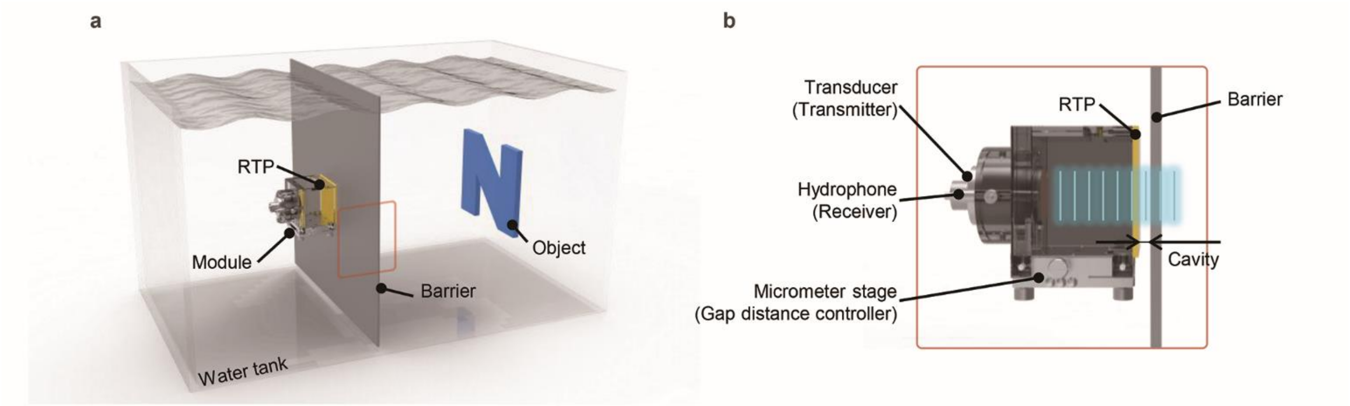

Supplementary Figure 8 | Current development: the modularized RTP for automated imaging. a, An experimental setup for automated barrier-through imaging using a modularized RTP. **b**, The magnified configuration of the modularized RTP. These configurations were drawn by SOLIDWORKS and rendered by KEYSHOT 10.

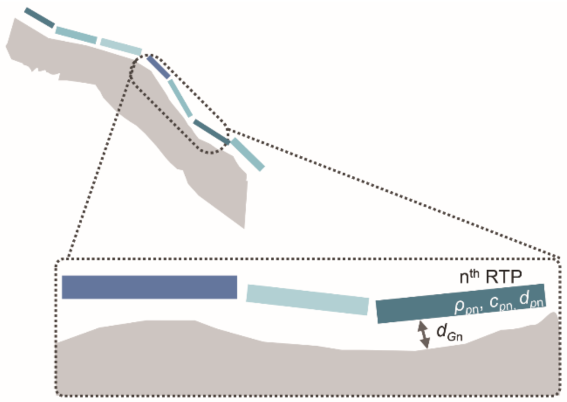

Supplementary Figure 9 | Piecewise segmented RTPs for thickness-varying barriers. Piecewise RTPs cover the entire surface of the barrier simultaneously featuring curvature and varying thickness. The RTP properties (ρ_{pn}, c_{pn}, and d_{pn}) are designed by Equation (1) in the main text.

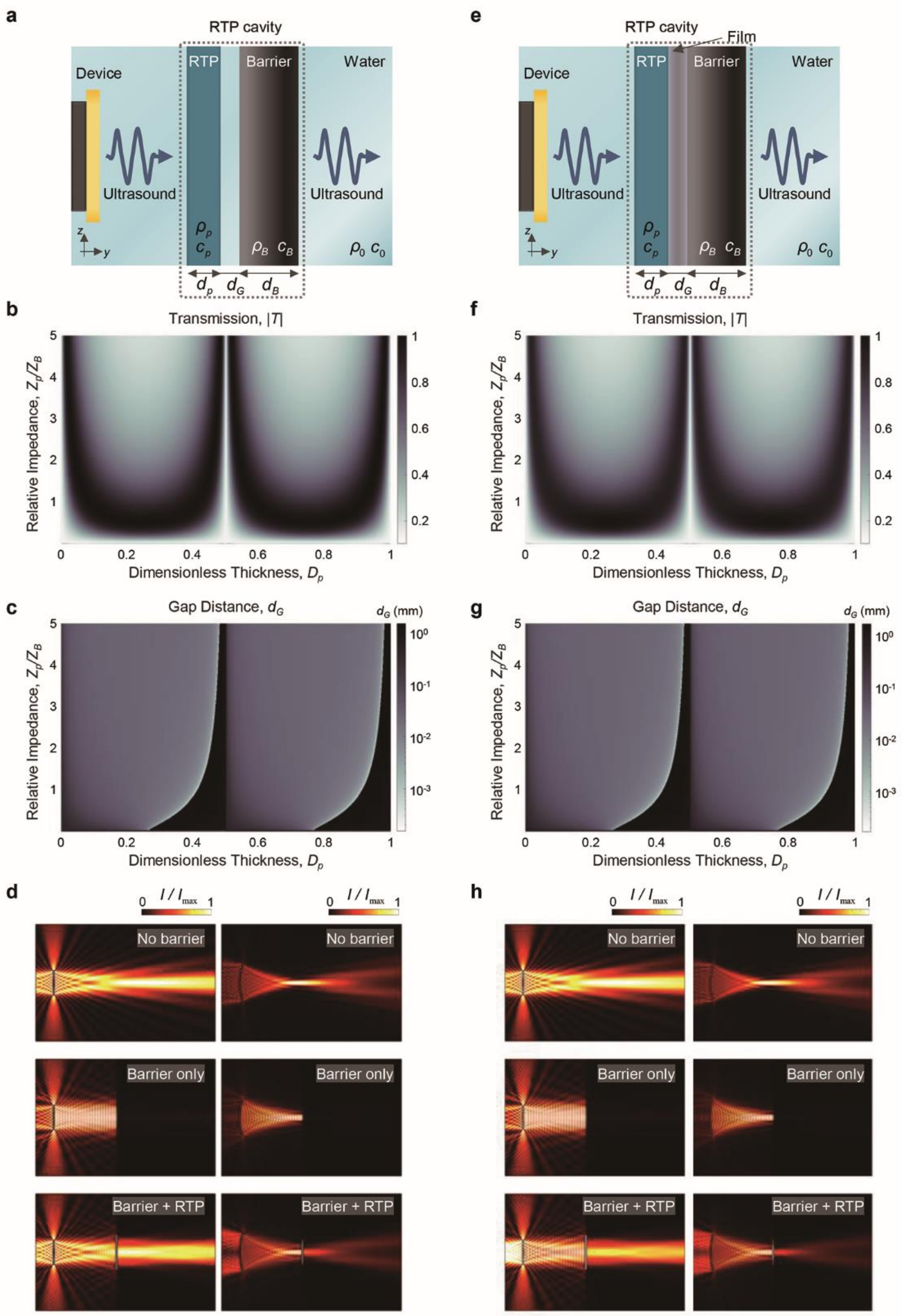

Supplementary Figure 10 | Film-employed RTPs to aid the gap distance control. Figures in **a-d** employ water gap as in the main text while those in **e-f** utilize the film made of ethyl vinyl acetate (28% acetate) whose material properties are density ρ_{EVA}=950 kg·m^{-3}, c_{EVA}=1650 m·s^{-1}, and impedance Z_{EVA}=1596 kPa·s·m^{-1}(=1.064Z_{Water}). **a**,**e**, A schematic configuration of the RTPs. **b**,**f**, Transmission contours. **c**,**g**, Gap distance contours. **d**,**h**, Ultrasonic beam transmission simulations. Quantitative data on the simulations are provided in Supplementary Table 5.

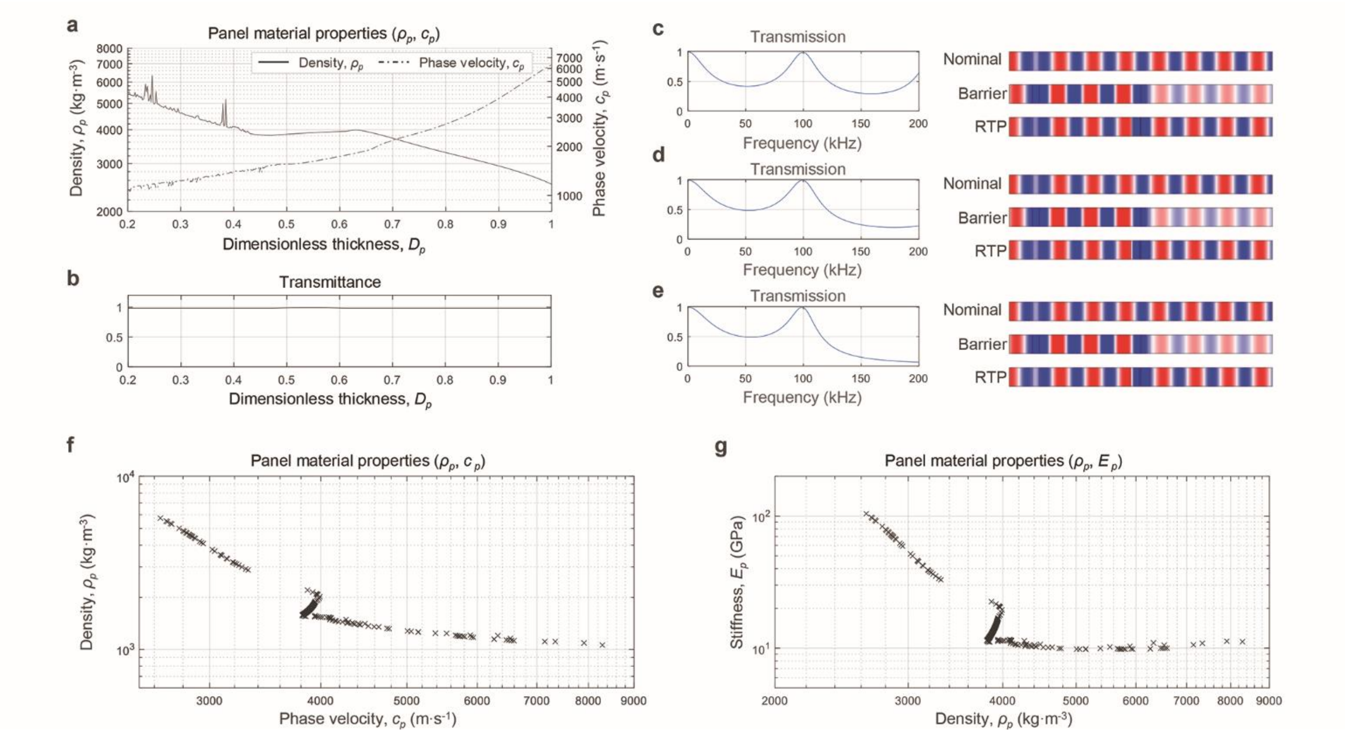

Supplementary Figure 11| RTPs for a fixed gap distance. RTPs for the full transmission for the given d_G by optimization. **a**, The material properties—density ρ_p by a solid grey line phase velocity c_p by a dash-single dot line—by the optimization. Here, 4-mm-thick aluminium was chosen both for a barrier and an RTP. **b**, The transmission coefficients by the optimized properties (density ρ_p, phase velocity c_p). **c-e**, The transmission curve (left) and the wave simulation (right) for three d_G's: **c**, d_G=0.2 mm, **d**, d_G=0.5 mm, and **e**, d_G=0.8 mm. The detailed RTP values are presented in Supplementary Table 6. **f-g**, Material property maps of the optimization results. **f**, The properties in the ρ_p-c_p plane. **g**, The properties in the ρ_p-E_p plane.

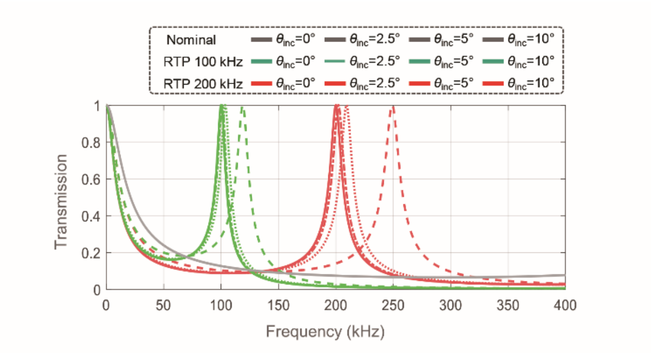

Supplementary Figure 12 | Oblique incidence effects to the RTP. The transmission curve of the normal and oblique incident cases. A 5-mm-thick steel barrier is considered here. As for the RTP, D_p= D_{B}, and Z_p/ Z_{B}=1 are chosen for 100 kHz and 200 kHz, respectively. The grey solid line denotes the transmission through the barrier only case for the normal incident wave. The green and red lines represent the barrier with the RTP at 100 kHz and 200 kHz, respectively. The incident angles are 0º, 2.5º, 5º and 10º.

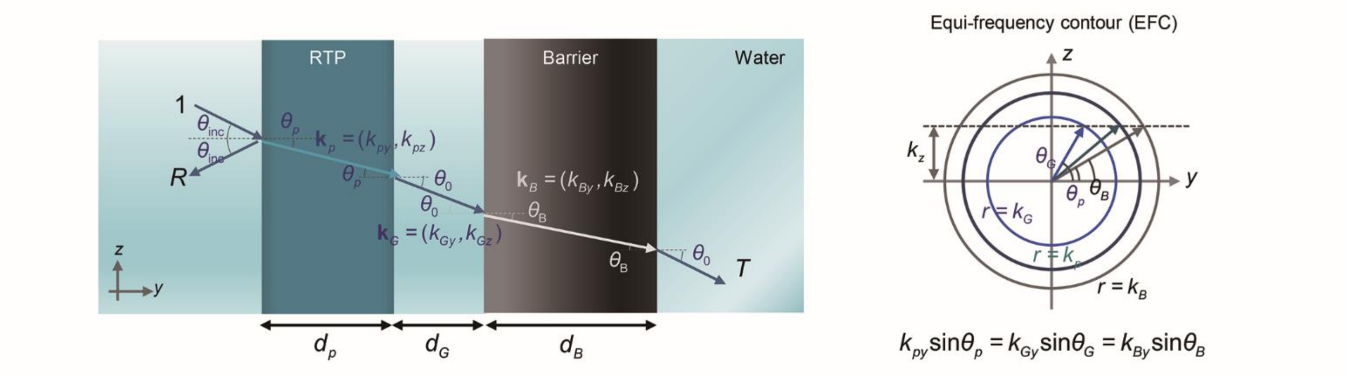

Supplementary Figure 13 | A schematic drawing for the RTP regarding obliquely incident wave. A schematic drawing for the wavevectors (left) and the corresponding equi-frequency contour (EFC).

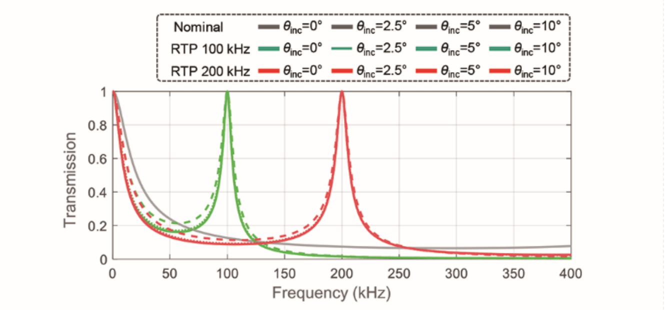

Supplementary Figure 14 | Examples of the modified RTPs corresponding to the incidence angle. Examples of the modified RTP system (d_G) corresponding to the incidence angles of 0º, 2.5º, 5º, and 10º. The configurations are all the same as those in Supplementary Fig. 12. We made modifications only to d_0 based on the wavevector calculation. The grey solid line denotes the barrier frequency dependency of the transmission for the normal incident wave to the barrier. The green and red lines represent the RTP installed systems for 100 kHz and 200 kHz, respectively.

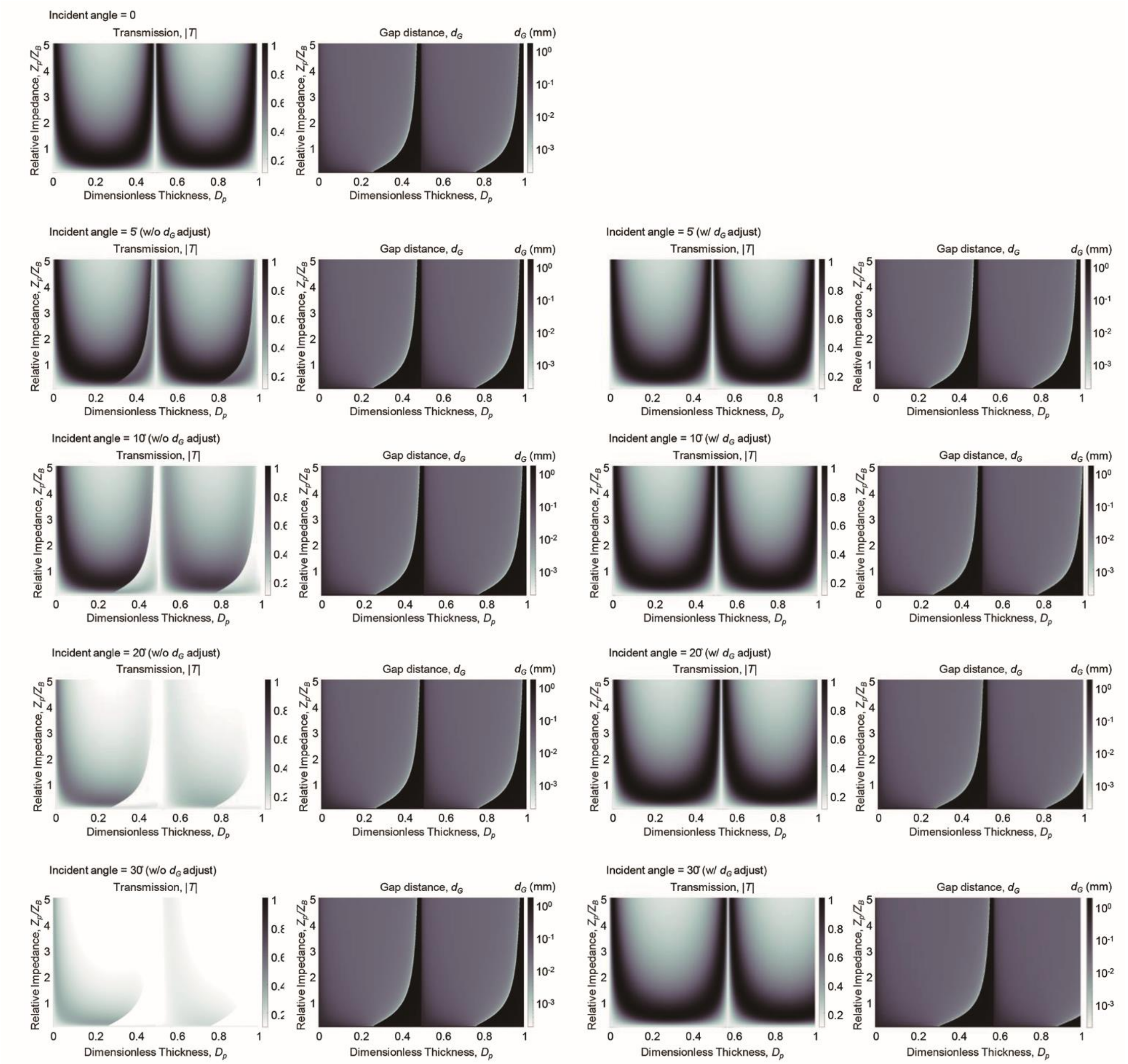

Supplementary Figure 15 | Examples of the modified RTP regarding the incidence angle. The transmission and the gap distance contours with and without the wavevector modification corresponding to the incidence angle. The incidence angle of 0º, 5º, 10º, and 20º are considered for a stainless steel barrier at 500 kHz. The results show that transmission decreases as the incidence angle increases for the cases without the correction. However, wavevector corrected cases show near-full transmission through the barrier at the given incidence angle.

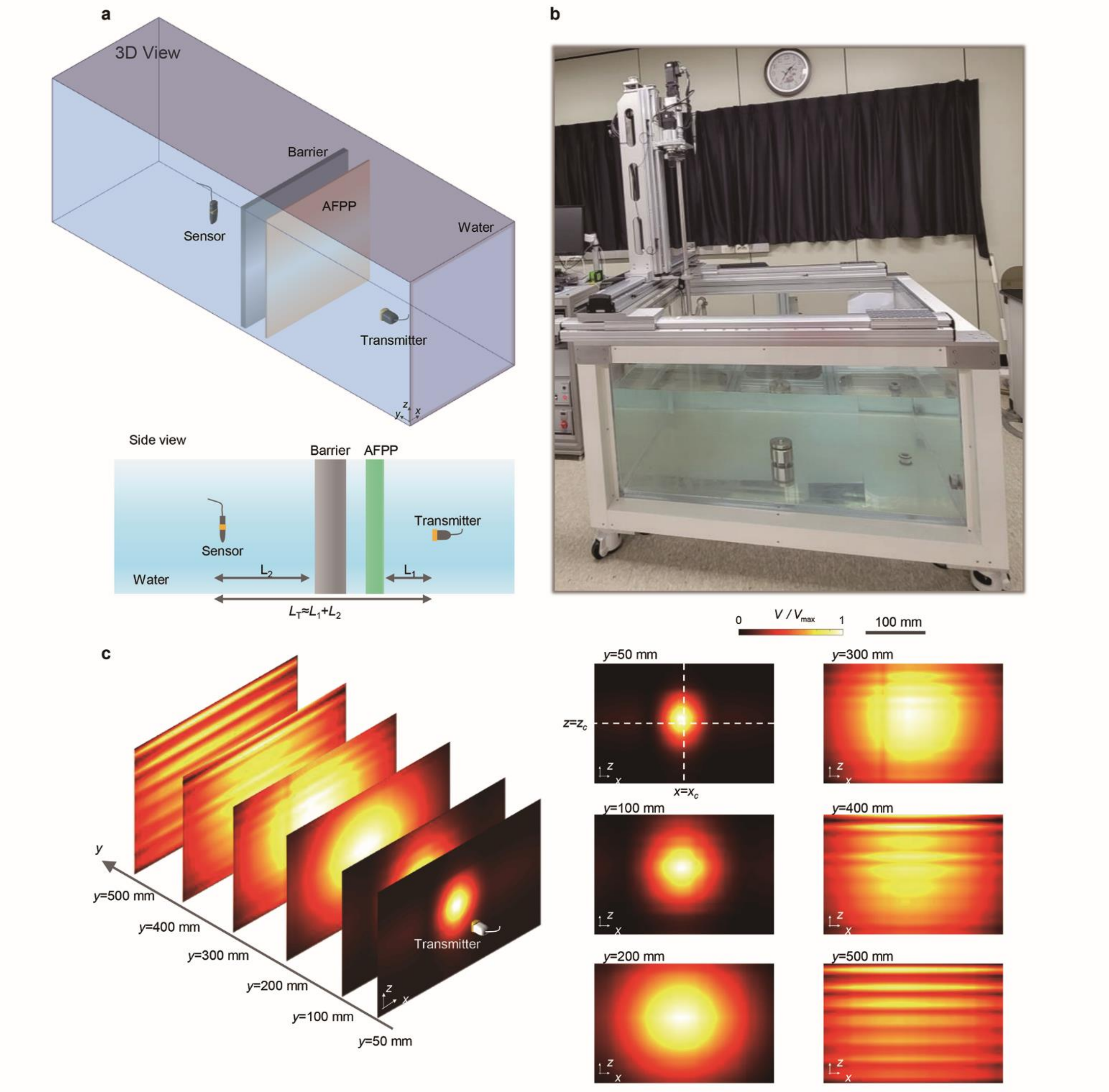

Supplementary Figure 16 | Experimental setup and the acoustic field. a, A schematic drawing of the experimental setup to measure the acoustic field. **b**, A photograph of the experimental setup and devices. **c**, Experimentally obtained transducer fields at distances of y = 50, 100, 200, 300, 400, 500 mm. A 100 mm scale bar is shown on the upper right.

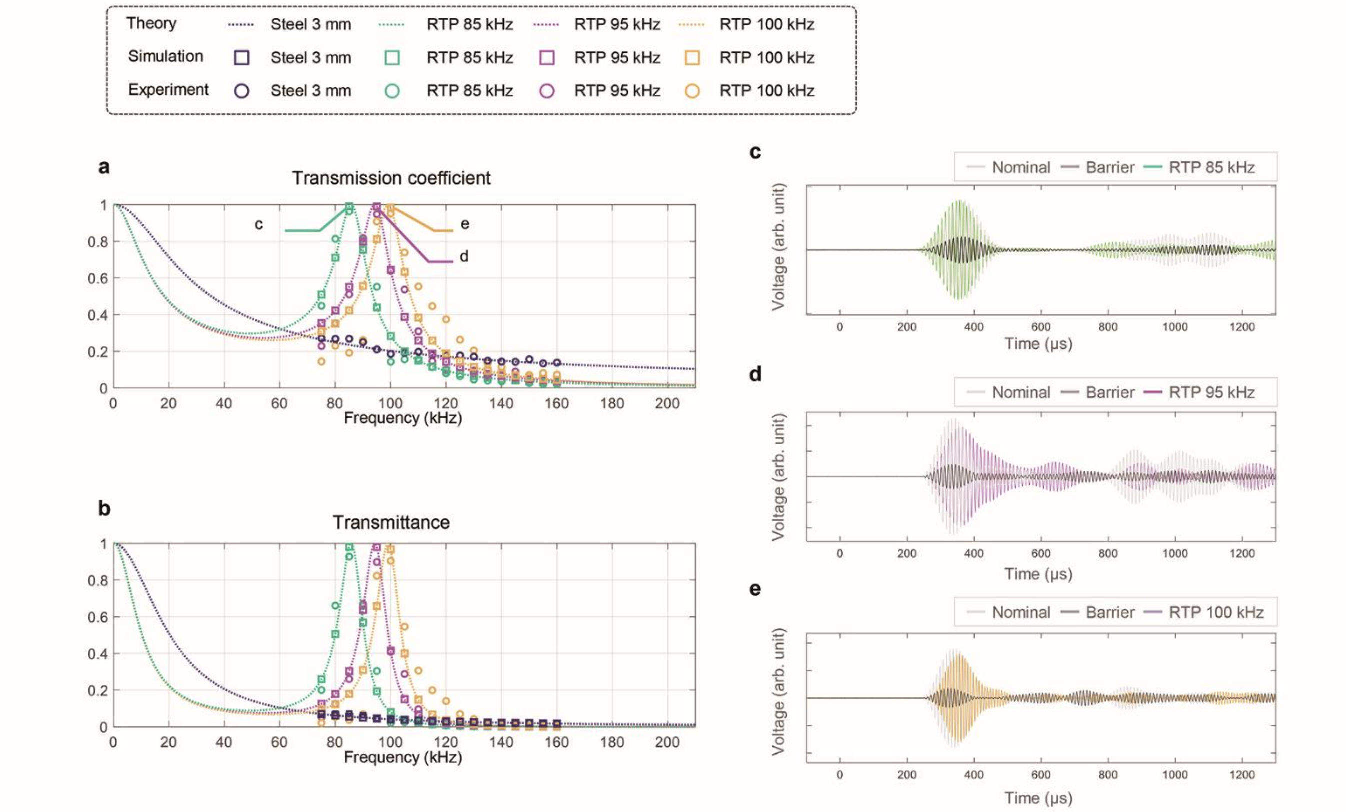

Supplementary Figure 17 | Experimental validation of the transmission. The frequency responses of the transmission coefficients were experimentally measured and compared both with the theoretical calculations and numerical results. **a**, Transmission curves. **b**, Transmittance curves. Time signal corresponding to the peak frequency of RTPs for **c**, 85 kHz, **d**, 95 kHz, and **e**, 100 kHz.

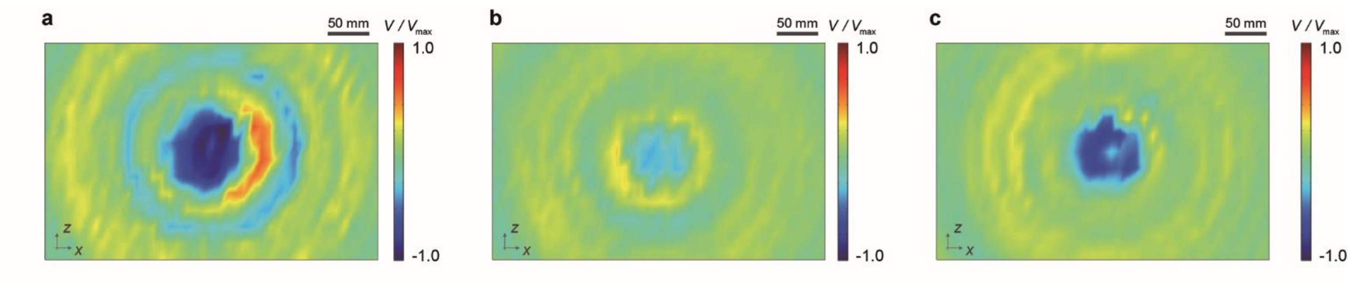

Supplementary Figure 18 | Experimentally scanned fields. Experimentally scanned fields (scale bar, 50 mm) for **a**, the nominal case, **b**, the barrier-only case (4-mm-thick steel), and **c**, the barrier with the RTP case. A scanning area with the size of 0.20×0.13 mm^2 is covered by 41 × 14 points. The mesh grid is 5×10 mm^2.

Supplementary Tables

Supplementary Table 1 | Designed RTPs for four different barriers.

Base material	d_B (mm)	Frequency (kHz)	Z_p/Z_B	D_p	d_p (mm)	d_G (mm)
Steel	1 mm	500	Z_{tu}/Z_{st} = 2.27	0.0361	0.481	0.055
			Z_{st}/Z_{st} = 1.00	0.0881	1.009	0.051
			Z_{al}/Z_{st} = 0.38	0.2321	2.925	0.030
Aluminium	1 mm	500	Z_{tu}/Z_{al} = 6.02	0.0121	0.161	0.161
			Z_{st}/Z_{al} = 2.65	0.0271	0.310	0.161
			Z_{al}/Z_{al} = 1.00	0.0791	0.997	0.149
Skull bone	1 mm	500	Z_{tu}/Z_{sk} = 17.31	0.0071	0.096	0.220
			Z_{st}/Z_{sk} = 7.62	0.0151	0.173	0.228
			Z_{al}/Z_{sk} = 2.88	0.0421	0.531	0.221
Aluminium	4 mm	700	Z_{tu}/Z_{al} = 6.02	0.4895	4.654	0.934
			Z_{st}/Z_{al} = 2.65	0.4775	3.905	0.931
			Z_{al}/Z_{al} = 1.00	0.4374	3.991	0.937

Barrier material: Steel (st), Aluminium (al), Skull bone (sk)
Panel material: Tungsten (tu), Steel (st), Aluminium (al)

Supplementary Table 2 | Detailed parameters of the imaging objects

Object	Base material	d_B (mm)	Reflection coefficient (%)		Width, Diameter (mm)	Structural similarity index		
			500 kHz	700 kHz		No barrier	Barrier+RTP	Difference (%)
Letter *N*	Steel	1 mm	99.2	99.6	80	0.6413	0.6222	2.98
Shape 1	Steel	1 mm	99.2	99.6	50	0.7225	0.7141	1.16
Shape 2	Steel	1 mm	99.2	99.6	30	0.7891	0.7790	1.28
Circle (Fig.3d)	Steel	2 mm	99.7	99.8	150	0.8196	0.7946	3.05
Circle (Fig.3e)	Steel	2 mm	99.7	99.8	150	0.8172	0.7930	2.96
Circle (Fig.3f)	Steel	2 mm	99.7	99.8	150	0.8154	0.7806	4.27

Supplementary Table 3 | Estimated centre locations by ultrasound images

Object location (mm)	Case	Estimated centre of the objects (mm)	Deviation (mm)
Left<br>(-100, 0)	No barrier	(-98.9, 1.7)	2.0
	Barrier+RTP	(-94.4, -3.6)	6.6
Middle<br>(0, 0)	No barrier	(0.0, 1.5)	1.5
	Barrier+RTP	(4.6, 3.5)	5.8
Right<br>(+100, 0)	No barrier	(99.5, 0.5)	0.7
	Barrier+RTP	(103.0, 0.3)	3.0

Supplementary Table 4 | Implementation parameters and transmittance from the simulation

Array type	Barrier	Material properties of RTP Density ($kg \cdot m^{-3}$)	Velocity ($m \cdot s^{-1}$)	Thickness (mm)	Distance d_G (mm)	Transmittance (%) Barrier only	Barrier+RTP	Transmittance enhancement (%)
Linear	Steel, 1 mm	8000	5723	1.00	0.051	1.57	91.01	5797
Focused						1.55	40.11	2588
Linear	Aluminium, 1 mm	8000	5723	0.31	0.161	12.12	99.82	824
Focused						11.86	78.75	664
Linear	Skull bone, 1 mm	8000	5723	0.17	0.228	31.30	99.95	319
Focused						30.30	75.32	249

Supplementary Table 5 | Simulation results for the RTPs employing different gap material

Gap material	Array type	Barrier	Material properties of RTP				Transmittance (%)		Transmittance enhancement (%)
			Density ($kg\cdot m^{-3}$)	Velocity ($m\cdot s^{-1}$)	Thickness (mm)	Distance d_G (mm)	Barrier only	Barrier+RTP	
Water	Linear	Steel, 1 mm	8000	5723	1.00	0.051	1.57	91.01	5797
	Focused						1.55	40.11	2588
Film	Linear	Steel, 1 mm	8000	5723	1.00	0.059	1.57	69.19	4401
	Focused						1.55	29.84	1925

Supplementary Table 6 | RTPs for given gap distance d_G's

	Distance d_G (mm)	Density ρ_p (kg·m^{-3})	Velocity c_p (m·s^{-1})	Transmittance coefficient (%)
Case 1	0.2	5484	5484	0.980
Case 2	0.5	3848	1632	0.988
Case 3	0.8	3286	2935	0.984

Supplementary Notes

Supplementary Note 1.

Research objective and the current development: modularized RTPs for automated barrier-through imaging system

Our goal is the development of a barrier-through imaging device for vessel accidents where information inside the vessel is highly required. Supplementary Figs. 7a and 7b present schematic illustrations. Divers acoustically observe inside the vessel using RTP and provide the information to the control tower.

To this end, we are working on improving TRL (technology readiness level) to reach real applications. One of our approaches is the modularized RTP shown in Supplementary Fig. 8a. We make the RTP as compact as possible. Our module consists of a transducer, hydrophone, and RTP combined with a micrometre stage. The micrometre controls the gap distance to adjust the previously calculated d_G for the given barrier information. For the next version, we are planning to automatically adjust d_G by extracting the barrier information. Note that barrier information can be calculated by frequency-dependent transmission and reflection coefficients. This RTP module will be installed on the outside of the vessel hull and provide ultrasonic images, as shown in Supplementary Fig. 8. We even can see-through hulls of different thicknesses easily by just changing the RTP. See the barrier-through imaging for different thicknesses and materials (Figs. 2 and 3 in the main text). Supplementary Fig. 6 also shows that our RTP can greatly increase the ultrasonic transmission through a wall, regardless of the material.

The targeting object is a simultaneously curved and thickness-varying barrier, as shown in Supplementary Fig. 9. We assume that the curvature and the variation in thickness are larger than the wavelength of the ultrasonic wave. This approach is also widely used in skull-through treatment in the biomedical field[1]. Piecewise RTPs cover the entire surface of the barrier. We theoretically find the RTP properties of an nth piece using Equation (1). We further cover the RTPs with the fixed gap distance in Supplementary Note 3.

Supplementary Note 2.

Discussion on the gap distance control and film-employed RTPs

This note covers the aspects of controlling the gap distance Since the gap distance is more significantly sensitive to the transmission than other parameters such as d_p, and d_B, one requires fine-tuning of the d_G to achieve high-quality barrier-through imaging.

To do so, as shown in Supplementary Fig. 8a, we conceived a compact-sized RTP. This RTP employs the feature size as compact as possible while still presenting reasonable transmission. By a series of numerical simulations, we observe that RTPs should have a larger aperture than 4-5λ. At 500 kHz, it corresponds to 15 mm. These RTPs, minimized in their size, are beneficial for the gap distance control over the entire RTP area. Errors—plate bending, local curvature, etc.—can be significantly alleviated compared to the equal-sized RTPs in Supplementary Fig. 8a.

The other way to facilitate the gap distance control is to replace the gap material with a solid film. Solid films can guarantee the gap distance. Supplementary Figs. 10a and 10e show the schematic configurations. We choose ethyl vinyl acetate (28% acetate), whose impedance is close to that of water. The material properties are as follows: density ρ_{EVA}=950 kg·m^{-3}, c_{EVA}=1650 m·s^{-1}, and impedance Z_{EVA}=1596 kPa·s·m^{-1}(=1.064Z_{Water}). We apply the same design procedure covered in Equations (7) to (12) in Methods under the assumption that that equation still holds for the film-employed RTPs. Supplementary Figs. 10b and 10f respectively show the transmission contours of the nominal (water-filled) RTP and film-employed RTP. Supplementary Figs. 10c and 10g present the corresponding gap distances. The film-employed RTPs present almost ideal transmission. We performed the beam transmission simulation using the RTPs to validate the film-employed RTP.

The target barrier was set to a 1-mm-thick steel barrier and the simulation setups are all the same as that in Supplementary Fig. 6. Supplementary Table 5 lists the details and Supplementary Figs. 10d and 10h depict the simulation results. The film-employed RTPs present extremely increased transmittance through the barrier. Although the increment is slightly lowered to some extent we observe the extreme contrast of the field. We can conclude that film-employed RTPs can be used as an alternative to the nominal one to facilitate gap distance control.

Supplementary Note 3.

RTPs for fixed gap distance and thickness

This note is devoted to covering the further understanding of the design of the RTP. RTPs have multiple solution sets as described in Supplementary Figs. 2a and 2b. In order to facilitate the RTP manipulation, in particular the distance d_G, we fixed d_G and numerically found the material properties (ρ_p, c_p) of the RTP. Optimization is used for the numerical method. To this end, we introduced the optimization formulation as

$$Minimize \left|\chi\left(\rho_p, c_p\right) - e^{2j\varphi_G}\right|^{\alpha}, \tag{1a}$$

$$subjected\ to\ \ T\left(\rho_p, c_p\right) \geq T_c\ . \tag{1b}$$

with the RTP condition in Equations (9-12) as

$$e^{2j\varphi_G} = \chi\left(\rho_p, c_p\right), \tag{2a}$$

$$T\left(\rho_p, c_p\right) = \frac{T_p T_B e^{-j\varphi_G}}{1 - R_p R_B e^{-2j\varphi_G}}, \tag{2b}$$

Supplementary Equation (1a) minimizes the magnitude of the difference between the left- and right-hand sides of Supplementary Equation (2a). An optimization parameter α was introduced to facilitate convergence. α was empirically chosen to be 0.05. The constraint was introduced to guarantee the transmission as in Supplementary Equation (1b). T_c was set to be T_c=0.99.

Supplementary Fig. 11 delineates the optimization results. Linearly distributed 500 d_G's cover the range from 0.2 mm to 1.0 mm. Here, the 4-mm-thick aluminium was chosen as a barrier (d_B = 4 mm), while the other dimensions are as follows: d_G=1 mm and d_p=4 mm. Supplementary Fig. 11a shows that the density and phase velocity converge to a single value for each d_G. The transmission coefficient described in Supplementary Fig. 11b is calculated by substituting the optimized values (ρ_p, c_p) into Supplementary Equation (2b). As shown in Supplementary Fig. 11b, the transmission over the whole range is close to 100%. This high transmission indicates the validity of the solutions.

One examines the solutions in the three different cases: d_G = 0.2, 0.5, 0.8 mm. The detailed information is listed in Supplementary Table 6. The left side in Supplementary Figs. 11c-e shows the transmission curve which fulfills the near-perfect transmission at the target frequency of 100 kHz. The left sub-figures show the numerical simulation at 100 kHz. From top to bottom, the nominal, barrier, and RTP cases are shown. It is clear for the RTP cases that transmission recovers close to 100%. These results verify the validity of the optimized values. Although we fixed d_G here, other parameters—d_p, ρ_p, and c_p—can be fixed instead of d_G if it facilitates the RTP functionality.

To see the tendency of material property combinations, properties are drawn on ρ_p-c_p plane and ρ_p-E_p

plane, respectively in Supplementary Figs. 11f, g. For Young's modulus calculation, the following equation is used:

$$E_p = 3\rho_p c_p^2 \left(1 - 2\nu_p\right), \tag{3}$$

under the assumption of the value of Poisson's ratio to be v_p=0.3, which is a typical value for the common metal. In Supplementary Fig. 11g, Young's modulus and density lie within the ranges of materials found in nature; from 10 to 100 GPa for Young's modulus, and from 2700 to 8300 kg·m^{-3} for density. It is seemingly materializable with materials in nature, however, material properties are located from the upper-left side to the lower-right side. This downward tendency is significantly opposite to that in the Ashby chart[2] where Young's modulus and density have an upward tendency. To realize those properties, metamaterials[3,4] breaking those limits have great freedom to choose the material properties and may provide a good solution to this problem. However, we do not present further since those designs are out of the scope of this research.

Supplementary Note 4.

RTPs for oblique incident waves

This note covers the RTPs for obliquely incident waves. The fully transmitted frequency varies regarding the angle. Supplementary Fig. 12 shows RTPs designed for 100 kHz and 200 kHz, respectively, for a 5 mm-thick steel barrier. As for the RTP, D_p= D_B and Z_r/Z_B=1 were used. The normal incident case (θ_{inc}=0°) presents its transmission peak at the exact target frequency. On the other hand, a shift of the transmission peak shift occurs for the oblique incidence. We present the transmission curve for varying angles in Supplementary Fig. 13; incident angles are θ_{inc}= 0, 2.5°, 5°, and 10°. The frequency peaks gradually shift to higher frequencies.

We considered the modified wavevectors for oblique incidence as shown in Supplementary Fig. 12. According to the assumption that all media are isotropic modified wavenumbers can be calculated as:

$$k_z = k_m \sin\theta_m \quad (m = G, p, B), \tag{5a}$$

$$\theta_m = \sin^{-1}(k_z / k_m)\,(m = G, p, B), \tag{5b}$$

$$\bar{k}_m = k_m \cos\theta_m \,(m = G, p, B). \tag{5c}$$

Supplementary Fig. 14 shows the results with a modified wavenumber. We assumed that the barrier only carries the longitudinal wave to ease the calculation. For all incidence angles, transmission peaks take place accurately at the target 100 kHz and 200 kHz, which implies that the proposed angle correction scheme is valid.

We further present the effect of incident angle in Supplementary Fig. 15. Though we changed the frequency to 500 kHz, this incident angle dependency observes a similar tendency to other sets of the barrier. Transmission becomes lower as the incident angle increases. This phenomenon can be explained in the same vein as in Supplementary Figs. 13 to 14. At incident angles of 30°, The highest transmission on the contour is below 0.36, even if they follow Equations (11) and (12) properly. However, if one modifies the wavenumber as Supplementary Equation (5), the transmission recovers up to 100% as observed in the figure on the third column in Supplementary Fig. 15. It is worthwhile to note that the dimensionless thickness of the panel d_p, becomes longer compared to the normal incidence. This can be attributed to the decreased wavenumber as shown in the EFC in Supplementary Fig. 13. Based on our observations, we concluded that although it is not possible to apply the same RTP for the normal incident wave, especially for large incident angle, RTPs can achieve full transmission even for obliquely incident waves by properly adjusting d_p,

Supplementary Note 5.

Experimental details and data for ultrasonic imaging

This note covers the preliminary experimental data for imaging. To minimize the reflections from the wall, a sufficiently large tank of 1.5×1.5 mm^2 (x×y) shown in Supplementary Fig. 16a was used. As for the barrier and RTP, plates the size of which is 800 mm × 600 mm (x×z) were used for a stable experiment.

An experimental procedure is as follows. A source signal, 10 cycles of the modulated sinusoidal function V_0×(1-cos($2\pi\eta t/20$))×sin($2\pi\eta t$) with the centre frequency η, was generated by the function generator (Agilent, 33250A) and then amplified by a power amplifier (T&C Power Conversion, AG1017L) and then sent to the wave source, transmitter (The Ultran Group, GS100-D25). This transmitter was so chosen to generate acoustic waves at 100 kHz. The transmitted wave was picked by a hydrophone (Teledyne Marine, TC 4034-1), then sent to a preamplifier (Stanford Research Systems, SR560), and finally sent to an oscilloscope (LeCroy, WaveRunner 104MXi-A). The hydrophone has stable measurements over the frequency range of our interest.

As shown in Supplementary Fig. 16a, a hydrophone picked up the wave signal in the scanning area, the size of which was 0.30×0.20 m^2. The scanning grid was set to 10×10 mm^2 so that the 31×21 points covered the entire region. The grid size of 10 mm (0.67λ) along the y and z axes, respectively, are less than a wavelength. This grid size allows one to capture sufficient details of the target. Instead of measuring the total pressure field, one constructed the wave field covering the scanning area by synthesizing the wave field on the centre line along y and z axes (i.e. $p(x, z_c)$, $p(x_c, z)$) as

$$p(x, y, z) = p(x, y, z_c) \cdot p(x_c, y, z), \quad (6)$$

with x_c=150 mm and z_c =100 mm. Supplementary Fig. 16c shows ultrasound wave fields measured at distances of y=50, 100, 200, 300, 400, and 500 mm. Each point in the contours represents the peak-to-peak pressure amplitude of the time signal. Based on those wave fields, we chose L_T =400 mm for the further test of RTP functionalities.

We validated the frequency response of the RTP experimentally. Supplementary Fig. 17 experimental results. A 3 mm-thick SUS 304 plate was placed between the transmitter and sensor (L_1=200 mm) as the barrier. Experimental results (circular marker) agree well with the theoretical results (dashed line) and simulation results (square marker). Transmission coefficients (as well as transmittance), defined as the transmitted amplitude ratio, were experimentally calculated over the frequency range (from 75 kHz to 160 kHz with a 5 kHz interval). While theoretical prediction was calculated by Equations (7) and (8), the simulation results were obtained through the process previously described in Methods in the main text. The nominal signal (light grey line) and barrier signal (dark grey line) are shown in Supplementary Figs. 17c to 17e points out the low transmission. The significant amplitude contrast between the nominal and

barrier case signal is triggered by the high impedance mismatched barrier. For instance, the transmitted wave energy at 100 kHz is less than 7%.

On the other hand, as for the RTP case, one designed RTPs for 85, 95, and 100 kHz are fabricated and implemented at the corresponding distance d_G (see Supplementary Table 6 for detailed information). Experimental results match well with the theoretical transmission curves as well as the simulation results. Little discrepancy (slight drop) between the experimental points, which may be attributed to experimental errors, does not belittle the drastically increased wave amplitude of RTPs. The raw signals of the transmission peak for each RTP are shown in Supplementary Figs. 17c to 17d with a green, magenta, and yellow color, respectively. The drastically recovered amplitude of the RTP signals is enough compared to that of the nominal signal represented by a light grey line, indicating solid validity of the transferring functionality of the RTPs.

The transmitted wave fields are measured as shown in Supplementary Fig. 18. To stably perform scans over the entire area, one used an optical microphone (Xarion, Eta 100 Ultra) as a sensor. 4-mm-thick aluminium was used as a barrier, and RTP made of a 4-mm-thick aluminium plate was installed at calculated d_G. A scanning grid with the size of 0.20×0.13 m^2 was chosen to sufficiently capture the wave field. A 5×10 mm^2 sized ($0.33\lambda \times 0.67\lambda$) mesh grid covers the total scanning area with 41×14 points to capture the detailed wave field. Supplementary Fig. 18a shows the wave field of the nominal case without barriers. As shown by the results the waves are concentrated at the centre. The barrier case in Supplementary Fig. 18b features the wave field of low transmitted energy. The RTP case in Supplementary Fig. 18c shows a high-intensity wave field comparable to the nominal case.

Supplementary Note References

1 Wang, T., & Jing, Y. Transcranial ultrasound imaging with the speed of sound-based phase correction: a numerical study. *Phys. Med. Biol.*, **58**, 6663 (2013).

2 Shercliff, H. R., & Ashby, M. F. Elastic Structures in Design. Encyclopedia of Materials: Science and Technology (Elsevier, Amsterdam, 2001).

3 Shen, C., Xu, J., Fang, N. X., & Jing, Y. Anisotropic complementary acoustic metamaterial for canceling out aberrating layers. *Phys, Rev. X*, **4**, 041033 (2014).

4 Park, C. I., Piao, C., Lee, H., & Kim, Y. Y. Elastic complementary meta-layer for ultrasound penetration through solid/liquid/gas barriers. *Int. J. Mech. Sci.*, **206**, 106619 (2021)
